# Nursing Students’ Views on Promoting Successful Breastfeeding in Sweden

**DOI:** 10.5539/gjhs.v6n5p63

**Published:** 2014-05-09

**Authors:** Zada Pajalic

**Affiliations:** 1 School of Health and Society, the PROCARE Group, The Network for Eating and Nutrition, Kristianstad University, Sweden; 2 Faculty for Midwifery, Oslo and Akershus University College of Applied Sciences, Norway

**Keywords:** breastfeeding, cultural acceptance, nursing students, promotion

## Abstract

Promoting breastfeeding is important work for health-care personnel in the Swedish context. This promotion is multifaceted and demands the ongoing development of knowledge and competence among both health-care personnel and patients.

The aim of the present study was to describe the nursing students’ perspectives on breastfeeding in Sweden. Data were obtained in the form of written reflections from nursing students (n=65) and examined using manifest content analysis. The results show that the factors of importance in promoting successful breastfeeding are information about breastfeeding’s benefits, traditions and cultural acceptance of the practice, and by government prohibition of infant formula. We conclude that knowledge about the benefits of breastfeeding needs to be prioritized continuously during education.

## 1. Background

A child has a right to adequate nutrition and access to a safe and nutritious diet (Hunsberger et al., 2013; Endresen & Helsing, 1995). More than 3,000 children die every day from infections transmitted through improperly and unhygienically prepared infant formula (Dalzell, Rogerson, &Martindale, 2010; Schubiger, Schwarz, & Tönz, 1997, 2003). In developing countries, mortality among newborn infants is 90 per 1,000, and most of these deaths can be associated with infant formulas.

Infant formulas feeding can be avoided, and exclusive breastfeeding is recommended during the first six months of life up to two years of age (Lawrence R. A. & Lawrence R. M., 2011; Riordan & Wambach, 2010; Trotter, 2004; Weimers & Gustafsson, 2000). The World Health Organization (WHO) has argued that governments should facilitate breastfeeding in every society and encourage each child’s development to his or her full potential (Schubiger et al., 1997). Scientific studies show that breastfeeding is beneficial in many ways. A recent study has shown that children who were breastfed for more than four months had between 20% and 25% lower risk of developing asthma before the age of eight (Doherty, Chopra, Nkonki, Jackson, & Persson, 2006; Haschke & van’t Hof, 2000; Turck et al., 2013). Children who were breastfed had, according to the study, better lung function than children who were not. Other research has shown that breast milk reduces the risk of ear infections, respiratory infections, diarrhoea, and urinary tract infections. Breastfed babies are also less likely to become obese as adults (Hofvander, 2005).

The WHO (Schubiger et al., 1997; Turck et al., 2013) and the American Academy of Pediatrics (AAP) (Eidelman & Feldman-Winter, 2005; Harder, Bergmann, Kallischnigg, & Plagemann, 2005; Kramer et al., 2001; Newton, 1971) have emphasized the value of breastfeeding for mothers and children alike. Both organizations recommend exclusive breastfeeding for the first six months of a child’s life. Authorities are working to minimize the risks of artificial infant formula (Abolyan, 2006; Gerard, 2001; Jonas et al., 2008), but infant formulas is not always the best choice. The last reported case of health problems caused by formula in Sweden demonstrates the complications that can arise from using infant formula: Swedish parents happened to mix a dangerous combination of infant formula that was too concentrated; it caused dehydration and cramping in a six-week-old infant, and the baby required intensive care for 36 hours (Dyson, McCormick, & Renfrew, 2005; Kohlhuber, Rebhan, Schwegler, Koletzko, & Fromme, 2008; Schubiger et al., 1997). The international code upheld by the WHO is meant to safeguard breastfeeding against marketing of infant formulas by companies that manufacture this product (Earle, 2002), and in Norway and Sweden, the marketing of infant formula is controlled (Silfverdal et al., 1997). Mother’s milk is the most natural food for an infant (Turck et al., 2013).

Breast milk offers many health benefits to the child (Nyqvist et al., 2012; Nyqvist & Kylberg, 2008). To start with, human milk contains active immune cells and antibodies that operate in the child’s body, providing resistance to various diseases and preventing the possible onset of allergies; breast milk is hygienic and easily accessible, promotes the mother-child connection, and is ideal nutritionally speaking (Kramer et al., 2001). Studies have shown, moreover, that breast milk can prevent necrotic bowel in premature newborns (Kramer et al., 2001; Liestøl, Rosenberg, & Walløe, 1988; Nelson, 2007). Breast milk gives the baby a more effective immune response than milk formula does; this has been demonstrated not only in developing countries but also in the West. Regarding mothers, breastfeeding is associated with better health through lessened bleeding after pregnancy and some protection against both breast cancer and ovarian cancer (Campbell & Jones, 1996; Dennis, 2002), as well as lower risk of developing breast, egg, and developing cancer of the reproductive organs as uterine cancer (Armstrong & Reilly, 2002; Saarinen & Kajosaari, 1995). Given that breastfeeding has been proved to promote the health of both mother and child, it is important to help mothers begin breastfeeding their children as early as possible (Flacking, Nyqvist, Ewald, & Wallin, 2003; Hannula, Kaunonen, & Tarkka, 2008).

Many women choose breastfeeding for the child’s sake (Nor et al., 2012). The literature review showed that breastfeeding policy and practice appear to be inadequate in most countries. Support (social, emotional, medical, or practical) is an important factor in a woman’s decision to breastfeed. Support measures at the national level that align with the WHO’s guidelines are crucial to the promotion of breastfeeding (McDonald, Henderson, Faulkner, Evans, & Hagan, 2010; Omer-Salim, Persson, & Olsson, 2007). Further research is needed about promoting breastfeeding and increasing knowledge of the practice. Highlighting professionals’ views on breastfeeding counseling may have long-range positive effects on breastfeeding frequency (Bier et al., 1997; Eidelman & Feldman-Winter, 2005). This study may contribute to new knowledge that may lead to increased breastfeeding rates nationally and internationally; by gaining insight into how nursing students in Sweden reason about successful breastfeeding.

## 2. Method

The nursing programme is three years long and after finishing it, students must apply for registration before they can work as registered nurses. During their second year of studies, students take a clinical course focusing on primary care, paediatrics, and obstetrics. The obstetrics curriculum focuses on normal pregnancy, childbirth, and breastfeeding. For this study, 92 students were invited to participate, and 65 chose to do so. Non-responsive analysis was not performed. Data was collected in written form; all the students received a paper with one question: *What do you consider success factors that promote breastfeeding in Sweden?* These answers were voluntary. The length of answers ranged from two words to two pages of handwritten text. The written text was analysed using qualitative analysis (Krippendorff, 2004). The text was first read all the answers to gain an overall impression of the content. The next step comprised counting repeated words and expressions. These were then structured into subcategories and subcategories were arranged in categories.

## 3. Ethical Considerations

The present study was performed in accordance with the WMA Declaration of Helsinki (WMA Declaration of Helsinki, 1964). All participants took part in the study after receiving detailed information about its aims and confirmation of their right to discontinue participation at any time without any consequences for them.

## 4. Results

The participants expressed the that breastfeeding’s benefits for children’s health are, ranging from allergy prevention to overall good health, not to mention a better mother-child connection, which is important for the future relationship. One participant expressed this perspective: “The breastfeeding mother’s breast is beautiful and the most perfect natural meal box”.

### 4.1 Information

Most students demonstrated targeted knowledge about breastfeeding’s advantages, such as the fact that breast milk provides stronger immune protection for child than infant formulas do. In Sweden the breastfeeding women have good access to means lactation from volunteers and from health and care centres.

In addition, many students reported that their beliefs about breastfeeding’s advantages are not based on evidence. They conclusively expressed the view that Swedish society informs citizens very well about the psychological and physiological advantages of breastfeeding for infants and their mothers. One student said the following: “It is, after all, a special moment that they mother and her baby share together. Comprehensive information received before and after childbirth about the positive effects of breastfeeding for both mother and child and about the practice’s importance in building up the relationship between mother and child. Individually personalized information received at hospital from a midwife and via media is also of great importance (Sweden is a welfare state that provides substantial information about breastfeeding). One student remarked, “The parents with children get good information about the advantages of breastfeeding as protection against infections, nutritional richness, closeness, and in establishing the link between child and parents”. “I believe that optimal information about how good and important it is to breastfeed, on TV and from health and care sector [is crucial to breastfeeding success]”. One student mentioned the significance of “good information at maternal health centres and childbirth units; good knowledge and easy access to personnel can support and help when things are not working with breastfeeding, and of course good tools, like a breast pump, [also help]”.

### 4.2 The Promotion of Breastfeeding

Many expressed that breastfeeding does for the child’s immune protection, nutrition, and nearness to the mother. “We know about the benefits of breastfeeding and the consequences of renouncing breastfeeding”. Participants described the promotion of breastfeeding in the health-care sector and in society as important. Promotion should focus on the facts that breast milk helps infants develop a strong immune defence in the first stage of life and is the most optimal diet for infants. Educating parents during pregnancy about the advantages of breastfeeding (and the drawbacks of choosing not to breastfeed) ensures that they know why it is important to breastfeed. One of the students remarked, “During my education I became aware of the benefits of breastfeeding, especially about breast milk’s nutritional content and the baby’s closeness to the mother so it extremely important to spread knowledge about breast milk’s benefits for infants’ health. I believe that breast milk is good for immunity, good for contact between mother and child, and that good information is available from health-care personnel”. Moreover, breast milk is free of charge and always available. And again, “To breastfeed is not only meal time, it is an important moment for closeness and to connect with the child. It is important for the child’s feeling of safety”.

### 4.3 Tradition and Culture

The majority of students agreed that culture in general and cultural acceptance of breastfeeding play important roles in terms of the possibility to breastfeed in public without inviting negative reactions. One student expressed the importance of culture this way: “The knowledge about breastfeeding you get from parents and friends is very important”. Students also highlighted such cultural aspects as tradition, research, and education as key factors in promoting breastfeeding. One said, “New mothers are supported by their surroundings, and they can breastfeed in public without shame”.

The position of women in various cultures and the father’s role influence successful breastfeeding. In Sweden, fathers have the right to paternity leave and to take care of their infants, supporting the mothers in breastfeeding by assisting in practical work at home, such as cooking, cleaning, changing diapers, and so on. Cultural and economic preconditions can affect success in breastfeeding, according to study participants, allowing the father’s role to be bigger than one might imagine if he can support the new mother in practical ways. One student described these preconditions in the following way: “Sometimes I believe that people think it is comfortable to not breastfeed and that it is easier with breast milk-substitutes. But breast-milk substitutes can be expensive and not as accessible as they need to be. Further, in many countries there is a problem finding water of good quality, and as a consequence, mixing a breast-milk substitute with contaminated water can be directly lethal for infants”.

But in some countries, the cultural and economic preconditions are not conducive to breastfeeding. For instance, in many places mothers cannot take much time away from their jobs after childbirth and do not have time to breastfeed. In other countries, mothers do not have access to the information about breastfeeding that is available in Sweden; this may lead mothers to not focus on breastfeeding. Further, it is important that families have access to enough literature about the subject. Good relations with a midwife and other health-care professional’s promotion. Sweden encourages mothers to breastfeed to a greater extent than do countries that have lower rates of breastfeeding.

### 4.4 Support for Breastfeeding Women

Support throughout the breastfeeding process is important so that parents can feel secure. Participants in this study indicated that support for women who choose to breastfeed takes several forms. First, a long maternity leave facilitates breastfeeding. Again, Sweden is a welfare state, so mothers are typically well-nourished and most have the ability to breastfeed. Society supports parents, especially mothers, by providing maternity leave and paternity leave, as well as advice and support if needed. One participant remarked, “New mothers have time to breastfeed when they are at home with their child”. In welfare countries in which such circumstances exist have a high percentage of healthy mothers, so there is low risk of transmitting diseases via breast milk to the infant. Many of these countries also have a strong tradition of breastfeeding; one student mentioned that in Sweden “many cafes and shops have areas that are more secluded, plus there are easy methods to use, such as special breastfeeding clothes so that the mother does not have to expose more of her chest than necessary”.

In addition, regular contact with a midwife is very important. In Sweden can breastfeeding mother get support from health-care personnel focusing on how to breastfeed. “Midwives will see that the new mothers breastfeed because they are updating their knowledge all time and know what is right. This contributes to more knowledge among mothers so they are better able to choose whether they will breastfeed or not”.

A parent’s educational level is also of importance; more knowledge means that they more easily accept information about breast milk’s positive effects on an infant’s health. Higher economic status and health status also influences mothers’ decisions to breastfeed.

### 4.5 Controlling the Marketing of Infant Formulas

Many participants expressed the opinion that marketing infant formulas should be forbidden; one said, “Marketing influences us whether we like it or not; it is OK to prohibit the marketing of infant formulas”. Such control is crucial because artificial infant formulas cause frequent complications, as mentioned earlier; they involve feeding with infant formulas and are associated with the increased prevalence of yeast infections in infants’ mouths. Marketing breast-milk substitutes should not be permitted.

### 4.6 Others’ Attitudes toward Breastfeeding

Many participants indicated that the attitudes of the people around a new mother play an important role in whether breastfeeding is taken for granted or viewed in another light. Even her relationship with her own mother can influence a new mother’s willingness to breastfeed. Further, respondents highlighted that breastfeeding is an option, it can be easier to breastfeed if a woman does not feel that she is obliged to. In fact, there is significant social pressure when it comes to expectations that a mother will breastfeed and great emphasis on breastfeeding and the value of the “natural” approach, as well as pressure to quickly return to work after birth. One participant expressed this view: “Breastfeeding is allowed in public places; it is not a limitation for breastfeeding women to breastfeed. Many in your circle of friends are breastfeeding; you’re talking about the advantages and disadvantages”. The strong focus on breastfeeding can develop a kind of positive group pressure, and this can create stress for new mothers. One respondent shared her experience: “Before I became a mother, I believed that everyone could breastfeed, but when I couldn’t manage it; this was a shock for me and a huge disappointment. Surroundings as unobtrusive places for breastfeeding, cosy lighting, and comfortable furnishings—play an important role. Access to maternal health centres, to assistance, and to monitoring, as well as the support received from personnel in the childbirth unit, can reduce the psychological stress of breastfeeding. The longer parental leave available in Sweden is crucial. Mothers are able to stay at hospital, and personnel give individually customized information to each mother so that she can feel self-confident. Closeness to the child is facilitated by the “*rooming into*” principle, which means that child and mother are together after childbirth. The mother’s access to experienced and skilled personnel is very important.

## 5. Discussion

This study shows that nursing students see promoting breastfeeding as an important for infant’s health. This supports the findings of Zwedberg (2010), who has examined mothers’ expectations of midwives. Women who did not have adequate support in terms of promotion experienced breastfeeding as more difficult, and their sense of being a “real” mother shifted to more negative attitudes if breastfeeding was terminated in an early stage. Zwedberg’s (2010) study indicates that mothers thought of breastfeeding as a “door opener” to their ultimate new mothering role and felt it was important that the midwife understand them and their needs. The study by Hofvander (2005) demonstrates that promotion with a focus on breastfeeding routines at maternity centres and hospitals increased the breastfeeding rate from 50% to 73% (US, 2007–2009). An absence of breastfeeding promotion resulted in a low breastfeeding rate (Hunt, 2006). Hunt (2006) has found that cultural norms regarding breastfeeding play an important role: if it is seen as taboo to breastfeed, many women may choose instead to bottle feed.

In the present study, the aim was to gain insight about how nursing students reason about breastfeeding because there is an expectation that practising nurses have to promote breastfeeding. Many of the participants’ responses seem to indicate that participants will promote breastfeeding in the future. That study also stressed the importance of monitoring and implementing “ten steps for successful breastfeeding” in all hospitals. The same conclusion was shown by recommendations that hospital have to adopt evidence-based practice to support breastfeeding in order to improve child health and to lower the rate of obesity (US, 2007–2009; Attard Montalto et al., 2010).

In the present study, it was highlighted that positive support from surrounding plays an important role in the success and frequency of breastfeeding. This confirms the results of Stewart-Knox et al. (2003), which stressed the influence of social norms, especially those that make it difficult for mothers to breastfeed (Stewart-Knox, Gardiner, & Wright, 2003). Hunt (2006) has also confirmed these results, describing the overall social acceptability of seeing a baby being bottle fed in public rather than seeing breastfeeding women, which is seen as taboo. On the other hand, success factors for breastfeeding women have been identified (O’Brien, Buikstra, & Hegney, 2008). The present study shows conclusively the great importance of promoting breastfeeding as a professional task of future registered nurses into their educational programs. Further, the promotion of breastfeeding needs to be continuous and updated regularly.
